# Frequency-Domain Detection for Frequency-Division Multiplexing QEPAS

**DOI:** 10.3390/s22114030

**Published:** 2022-05-26

**Authors:** Xiang Chen, Hao Liu, Mai Hu, Lu Yao, Zhenyu Xu, Hao Deng, Ruifeng Kan

**Affiliations:** 1 Jinlin Institute of Technology, Nanjing 211169, China; xchen@aiofm.ac.cn; 2 Hefei Institute of Physical Science, Chinese Academy of Sciences, Hefei 230031, China; haoliu@aiofm.ac.cn (H.L.); humai@aiofm.ac.cn (M.H.); lyao@aiofm.ac.cn (L.Y.); zyxu@aiofm.ac.cn (Z.X.); hdeng@aiofm.ac.cn (H.D.); 3 University of Science and Technology of China, Hefei 230026, China

**Keywords:** photoacoustic spectroscopy, quartz tuning fork, frequency-division multiplexing, frequency-domain signal

## Abstract

To achieve multi-gas measurements of quartz-enhanced photoacoustic spectroscopy (QEPAS) sensors under a frequency-division multiplexing mode with a narrow modulation frequency interval, we report a frequency-domain detection method. A CH_4_ absorption line at 1653.72 nm and a CO_2_ absorption line at 2004.02 nm were investigated in this experiment. A modulation frequency interval of as narrow as 0.6 Hz for CH_4_ and CO_2_ detection was achieved. Frequency-domain 2*f* signals were obtained with a resolution of 0.125 Hz using a real-time frequency analyzer. With the multiple linear regressions of the frequency-domain 2*f* signals of various gas mixtures, small deviations within 2.5% and good linear relationships for gas detection were observed under the frequency-division multiplexing mode. Detection limits of 0.6 ppm for CH_4_ and 2.9 ppm for CO_2_ were simultaneously obtained. With the 0.6-Hz interval, the amplitudes of QEPAS signals will increase substantially since the modulation frequencies are closer to the resonant frequency of a QTF. Furthermore, the frequency-domain detection method with a narrow interval can realize precise gas measurements of more species with more lasers operating under the frequency-division multiplexing mode. Additionally, this method, with a narrow interval of modulation frequencies, can also realize frequency-division multiplexing detection for QEPAS sensors under low pressure despite the ultra-narrow bandwidth of the QTF.

## 1. Introduction

Quartz-enhanced photoacoustic spectroscopy (QEPAS) [1,2,3,4,5] is now widely employed in gas measurements due to its high sensitivity, zero baseline, and excellent anti-disturbance performance. With the implementation of QEPAS, a modulated laser beam is collimated and then focused between the prongs of a quartz tuning fork (QTF) [6,7,8]. Acoustic microresonators with optimal parameters and configurations are commonly employed to increase sensor sensitivity [9,10,11]. With the laser absorbed by target gases, an acoustic wave is generated through non-radiative relaxation and a piezoelectric current is generated by the mechanical vibration of the QTF prongs, which is then converted to an output voltage by a trans-impedance amplifier. With a lock-in amplifier, the photoacoustic signal, usually in its second harmonic (2*f*) regime, can hence be demodulated [12,13].

To detect multi-component gas mixtures with QEPAS, time-division multiplexing technology has been commonly adopted to regulate multiple lasers of different central wavelengths [14,15]. Diode lasers are controlled via switching modules to direct a single beam to the acoustic detection module separately. The response time would consequently degrade with the increase of gas species. An acoustic detection module based on multiple QTFs [16] or a QTF combining different resonate frequencies [17] can realize simultaneous gas detection. However, the alignment and processing technology of the special QTFs will be complicated. A frequency-division multiplexing QEPAS sensor has thus been demonstrated for accurate measurements of CH_4_ and CO_2_ with an ordinary acoustic detection module [18]. To eliminate interferences between 2*f* signals retrieved by lock-in amplifiers, the modulation frequencies must be set with a sufficient interval. Nevertheless, the frequency bandwidth of the QTF is usually several hertz and will decrease sharply at low pressure [19,20]. For example, the frequency bandwidth of the QTF is 2.5 Hz at atmospheric pressure [21]. For precise dual gas detection at atmospheric pressure under the frequency-division multiplexing mode, a 2.5-Hz difference of modulation frequencies is still within the bandwidth and will lead to interference-free 2f components. However, compared with the 2.5 Hz difference, the amplitudes of the QEPAS signal will increase substantially with a narrower difference, according to the QTF response, since the modulation frequencies will be closer to the resonant frequency of the QTF. For the fixed bandwidth of the QEPAS sensor, more gases can be detected simultaneously with more lasers operating under the frequency-division multiplexing mode if the frequency interval is narrower. Moreover, for gas detection of more than three species under the frequency-division multiplexing mode, the required bandwidth of the QTF should be larger than 5.0 Hz if the frequency difference is 2.5 Hz. Additionally, the required bandwidth will rise linearly with the increase of gas species. As a result, the required bandwidth will exceed the bandwidth of QTF for multi-gas detection with a relatively wide interval of modulation frequencies under the frequency-division multiplexing mode. Hence, a wider difference in modulation frequencies will cause a serious decline in the QEPAS signals for gas detection of more than three species at atmospheric pressure due to the limited bandwidth of the QTF. Commonly, the bandwidth of the QTF will decrease sharply at low pressure; the bandwidth of the QTF decreases to 0.9 Hz at 50 Torr [21]. For gas detection with low pressure, the 2.5 Hz difference already exceeds the 0.9-Hz bandwidth of the QTF and the QEPAS signals will decrease significantly. Hence, the wide difference in previous papers shows that precise gas detection under the frequency-division multiplexing mode at low pressure cannot be realized.

In this work, we introduce a frequency-domain detection method for frequency-division multiplexing QEPAS sensors with a narrow modulation frequency interval. A CH_4_ absorption line at 1653.72 nm and a CO_2_ absorption line at 2004.02 nm were investigated in this experiment. Modulation frequencies of 16,356.18 and 16,356.78 Hz for CH_4_ and CO_2_ detection with an interval of as narrow as 0.6 Hz were employed. Frequency-domain 2*f* signals were obtained with a resolution of 0.125 Hz using a real-time frequency analyzer. With the employment of multiple linear regressions [22], precise gas detection was achieved despite the serious interferences between the 2*f* components.

## 2. Principle of QEPAS

The QEPAS signal can be expressed as

(1) S=K⋅Q⋅P⋅c⋅α(v)⋅ε

where *K* is the sensor constant, *Q* is the quality factor of QTF, *P* is the laser power, *c* is the target gas mole fraction, *α*(*v*) is the absorption coefficient, and *ε* is the conversion efficiency. For gas detection of CH_4_ and CO_2_ with the frequency-division multiplexing mode, the detected frequency-domain 2*f* components can be treated as the superposition of the 2*f* components of CH_4_ and CO_2_

(2) $$S_{2f}=S_{CH4\_d}+S_{CO2\_d}$$

where *S_CH*4*_d_* is the CH_4_ signal and *S_CO*2*_d_* is the CO_2_ signal. Since the 2*f* components are proportional to gas mole fractions, Equation (2) can be expressed as

(3) $$S_{2f}=k_{1}\cdot S_{CH4\_ref}+k_{2}\cdot S_{CO2\_ref}$$

where *S_CH*4*_ref_* is a reference signal of CH_4_ with a known concentration, *S_CO*2*_ref_* is a reference signal of CO_2_ with a known concentration, *k*_1_ is the scale factor of CH_4_, and *k*_2_ is the scale factor of CO_2_. Hence, mole fractions of the experimental 2*f* components can be retrieved with the reference 2*f* components.

## 3. Instrument Setup

**Figure 1:**
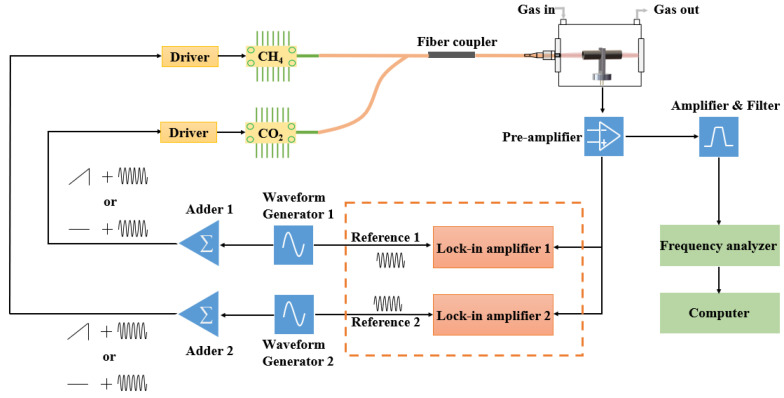
Instrument setup.

Figure 1 shows the instrument setup for a frequency-division multiplexing QEPAS sensor with the frequency-domain detection method. DFB lasers (NEL), operated at 1654 and 2004 nm, are selected as light sources for CH_4_ and CO_2_ detection. A CH_4_ absorption line at 1653.72 nm and a CO_2_ absorption line at 2004.02 nm are investigated in this experiment. The DFB lasers are controlled by home-made temperature and current drivers. Frequency-division multiplexing signals are generated with waveform generators (SDG1032X, Siglent, Shenzheng, China). The modulated laser beams are combined and then focused between the prongs of the QTF. Photoacoustic current from the QTF is converted to an output voltage by a trans-impedance amplifier and is then amplified again and filtered to enhance the signal-to-noise ratio. The center frequency of the bandpass filter is located at 32,713 Hz, and its bandwidth is compressed to 650 Hz. Output signals from the filter are processed by a real-time frequency analyzer (RSA5115B, Tektronix, OR, USA) with a sampling rate of 200 MHz and a resolution of 16 bits. To implement a comparison experiment, lock-in amplifiers (RS865A, Stanford Research Systems, Sunnyvale, CA, USA) are utilized to retrieve 2*f* components from the photoacoustic signals at different frequencies. The frequency-domain and time-domain signals are recorded and analyzed by a computer.

## 4. Experimental Results

### 4.1. Frequency Response of QTF

**Figure 2:**
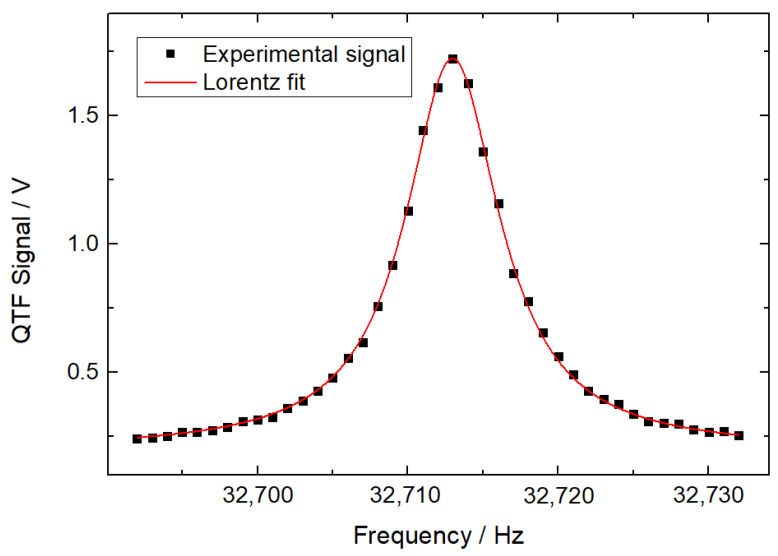
Frequency response of the QTF.

The frequency response of the QTF is shown in Figure 2. Sinusoidal signals of different frequencies were generated via a function generator to measure the frequency response of the QTF. The frequency of the sinusoidal signals was scanned from 32,688.00 to 32,732.00 Hz with a step of 1.00 Hz. Peak values of output signals from the QTF were fitted using the Lorentz profile-based nonlinear least square method. The calculated resonant frequency and response bandwidth of the QTF were 32,712.96 and 7.65 Hz, respectively, which led to a quality factor of 4267. To demonstrate the frequency-domain detection method for frequency-division multiplexing QEPAS sensors, modulation frequencies of 16,356.18 Hz for CH_4_ detection and 16,356.78 Hz for CO_2_ detection were selected. The corresponding second harmonic frequencies were located at 32,712.36 and 32,713.56 Hz with a 1.20-Hz interval.

### 4.2. Modulation Signal Optimization

**Figure 3:**
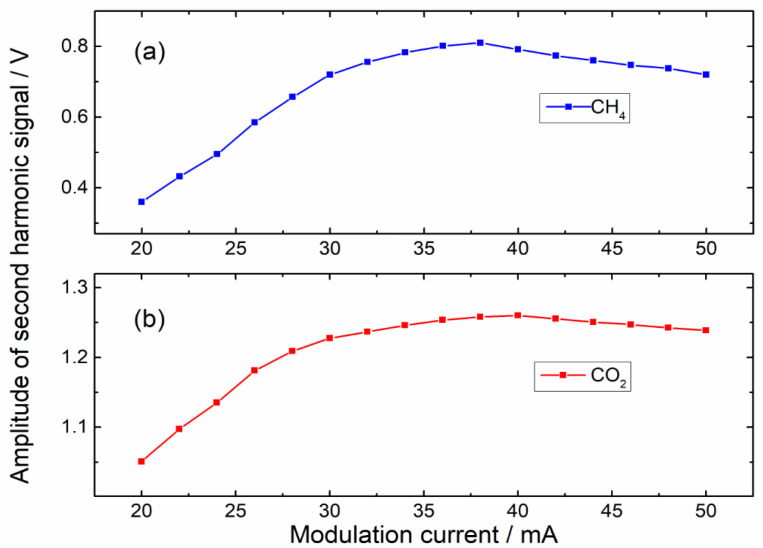
Amplitudes of second harmonics of CH_4_ and CO_2_, corresponding to different modulation currents. (**a**) CH_4_; (**b**) CO_2._

Since the laser modulation coefficient varied with the modulation current directly, the modulation current should be optimized to improve the amplitudes of 2*f* components. Two DFB lasers were operated alternately, and the sawtooth ramp signal of 0.1 Hz, superimposed with a sinusoidal modulation signal, was used to modulate laser injection currents. Gas samples of CH_4_ and CO_2_ were injected into the photoacoustic cell alternately with a flow rate of 50 mL/min. Lock-in amplifiers were utilized to retrieve 2*f* signals, and the amplitudes of 2*f* signals at various modulation currents are depicted in Figure 3. According to the result, modulation currents of 38 mA for CH_4_ and 40 mA for CO_2_ were selected in the subsequent experiments.

### 4.3. Time-Domain 2f Signals with Frequency-Division Multiplexing Mode

With the implementation of the frequency-division multiplexing technique, appropriate intervals between modulation frequencies should be determined to optimize the detection limits and avoid interferences. The frequency bandwidth of the QTF is relatively narrow and will decrease sharply at low pressure. Hence, a smaller modulation interval can enable more lasers to work at the same time under the frequency-division multiplexing mode. However, the small modulation interval will cause serious interferences between 2*f* components of target gases.

**Figure 4:**
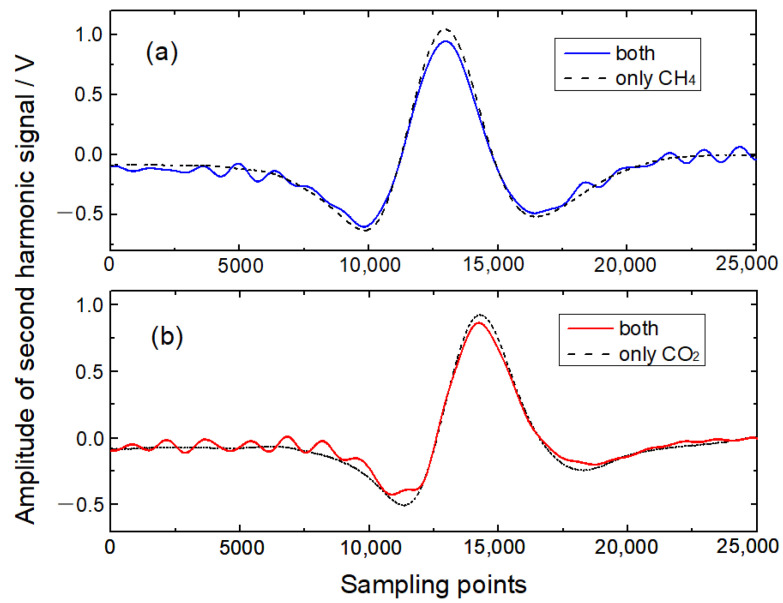
Time-domain 2*f* signals with the frequency-division multiplexing mode. (**a**) CH_4_; (**b**) CO_2._

In this experiment, modulation frequencies of 16,356.18 Hz for CH_4_ detection and 16,356.78 Hz for CO_2_ detection were selected. Modulation signals superimposed with sawtooth ramp signals of 0.1 Hz were used to modulate laser injection currents with the frequency-division multiplexing mode. A gas mixture of 300 ppm CH_4_ and 1600 ppm CO_2_ was injected into the photoacoustic cell with a flow rate of 50 mL/min. Lock-in amplifiers were used to demodulate the photoacoustic signals simultaneously, and the time constant was set as 300 ms. As depicted in Figure 4, the time-domain 2*f* signals are plotted with solid lines when two lasers operate simultaneously and with dotted lines when only a single laser operates. Serious interferences are observed between time-domain 2*f* signals with a 1.20-Hz interval of demodulation frequencies. The amplitudes of non-absorption wings are 8.1% and 11.1% of the amplitudes of time-domain 2*f* signals of CH_4_ and CO_2_. The corresponding signal-to-noise ratios are 42 and 39, which can lead to weakened detection limits. Furthermore, the interferences could change with different concentrations of gas mixtures. Thus, the time-domain 2*f* signals demodulated by lock-in amplifiers can hardly satisfy the demand for precise gas detection under the frequency-division multiplexing mode with narrow intervals of modulation frequencies.

### 4.4. Frequency-Domain Analysis

**Figure 5:**
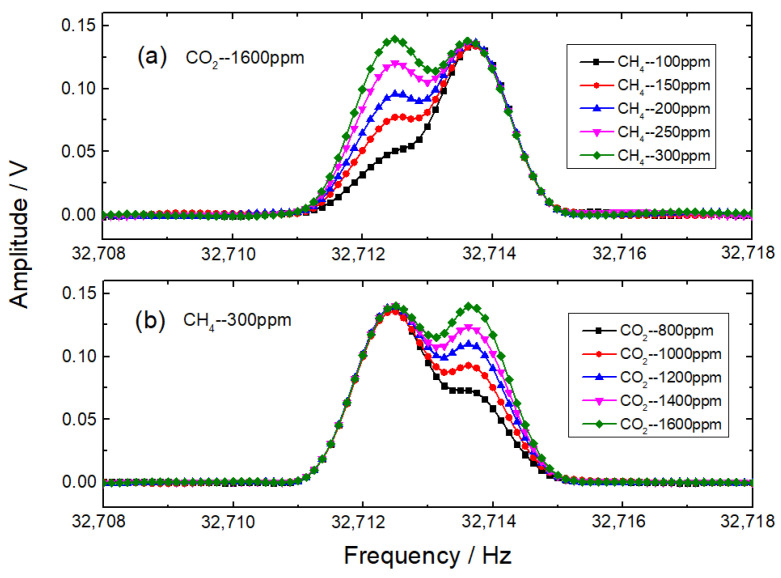
Frequency-domain 2*f* signals of different gas mixtures. (**a**) First group; (**b**) second group.

To examine the frequency-domain features of photoacoustic signals, gas mixtures of different concentrations were injected into the photoacoustic cell alternately, with a flow rate of 50 mL/min. For the first group of gas mixtures, the CO_2_ mole fraction was fixed at 1600 ppm, while CH_4_ mole fractions ranged from 100 ppm to 300 ppm with a step of 50 ppm. For the second group of gas mixtures, the CH_4_ mole fraction was fixed at 300 ppm, while CO_2_ mole fractions ranged from 800 to 1600 ppm with a step of 200 ppm. Modulation frequencies of 16,356.18 Hz for CH_4_ detection and 16,356.78 Hz for CO_2_ detection were selected, and sawtooth ramp signals were replaced with bias signals. A real-time frequency analyzer was utilized to process raw photoacoustic signals of different gas mixtures with a sampling time of 8 s. The frequency-domain 2*f* signals were 10 times averaged, and the frequency-domain resolution was 0.125 Hz. Figure 5 demonstrates the experimental results of different gas mixtures. The frequency-domain 2*f* signals of CH_4_ and CO_2_ mutually overlap, and gas concentrations cannot be directly retrieved from the spectra. Since the experimental signals can be treated as the superposition of the frequency-domain 2*f* signals of CH_4_ and CO_2_, multiple linear regressions can be employed to eliminate interferences.

**Figure 6:**
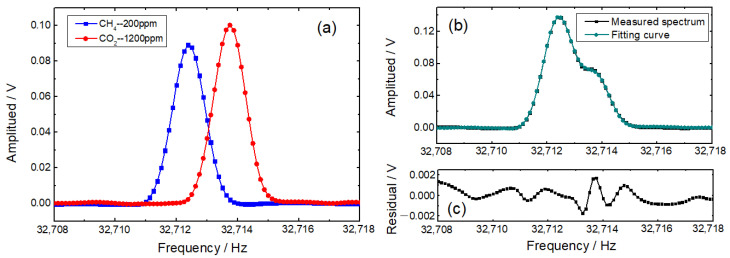
(**a**) Frequency-domain 2*f* signals of 200 ppm CH_4_ and 1200 ppm CO_2_; (**b**) a demonstration of multiple linear regression with the gas mixture of 300 ppm CH_4_ and 800 ppm CO_2_; (**c**) fitting residuals.

Standard gas samples of 200 ppm CH_4_ and 1200 ppm CO_2_ were injected into the photoacoustic cell successively, with a flow rate of 50 mL/min. As depicted in Figure 6a, the frequency-domain 2*f* signals of CH_4_ and CO_2_ are measured individually and are utilized as the basics in the application of multiple linear regressions. Figure 6b demonstrates an excellent fitting result of a gas mixture of 300 ppm CH_4_ and 800 ppm CO_2_, which is confirmed by the negligible <1.5% difference shown in Figure 6c.

**Figure 7:**
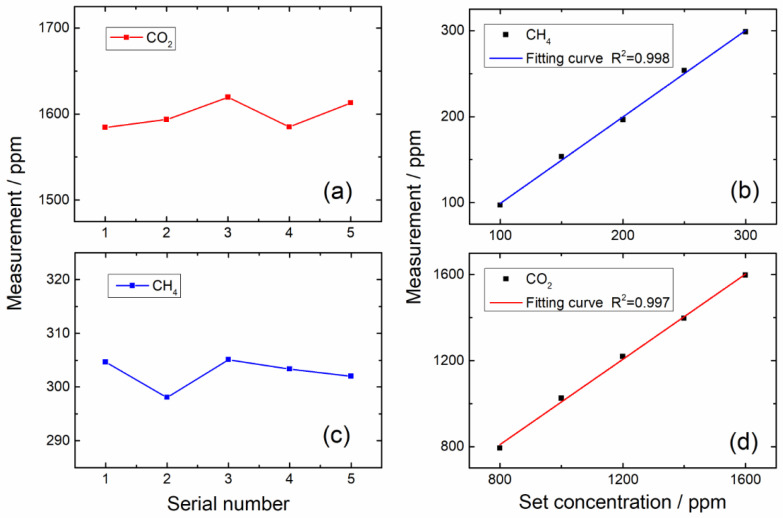
Best-fit mole fractions of (**a**) CH_4_ and (**c**) CO_2_ retrieved with multiple linear regressions. Best-fit mole fractions of (**b**) CH_4_ and (**d**) CO_2_ as the function of calibrated gas samples.

For the first group of gas mixtures, the calculated mole fractions of CO_2_ are shown in Figure 7a; the deviations of CO_2_ measurements are −1.3%, −0.5%, 1.6%, 1.3%, and 1.1% respectively. As shown in Figure 7b, there is a good linear relationship between the best-fit mole fractions of CH_4_, obtained by the multiple linear regression method, and the calibrated gas samples, with a linear correlation coefficient of 0.998. For the second group of gas mixtures, the calculated mole fractions of CH_4_ are shown in Figure 7c; the deviations of CH_4_ measurements are 2.3%, −0.9%, 2.5%, 1.7%, and 1.0%, respectively. As shown in Figure 7d, there is a good linear relationship between the best-fit mole fractions of CO_2_, obtained by the multiple linear regression method, and the calibrated gas samples, with a linear correlation coefficient of 0.997. Based on the results of multiple linear regressions with various gas mixtures, small deviations and good linear relationships for gas detection can be observed under the frequency-division multiplexing mode with a narrow interval of modulation frequencies.

**Figure 8:**
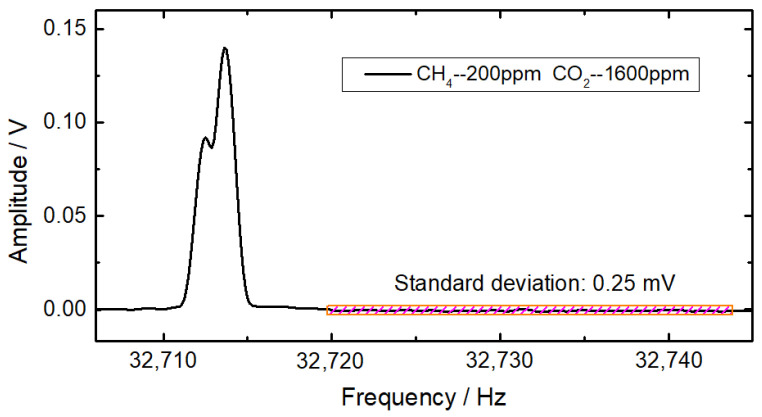
Evaluation of detection limits.

To evaluate the detection limits of CH_4_ and CO_2_, the gas mixture of 200 ppm CH_4_ and 1600 ppm CO_2_ was injected into the photoacoustic cell with a flow rate of 50 mL/min, and the profile of the frequency-domain QEPAS signal was averaged over 50 times to enhance the signal-to-noise ratio. The peak values of the frequency-domain 2*f* signals of CH_4_ and CO_2_ were determined with multiple linear regressions. As depicted in Figure 8, the standard deviation of the background is 0.25 mV, which corresponds to the signal-to-noise ratio of 332 for CH_4_ and 551 for CO_2_. As a result, the minimum detection limits of CH_4_ and CO_2_ are 0.6 ppm and 2.9 ppm, respectively.

## 5. Conclusions

Since the frequency bandwidth of the QTF is relatively narrow and will decrease sharply at low pressure, a narrower interval of modulation frequencies can enable more lasers to work at the same time with the frequency-division multiplexing mode for QEPAS sensors. However, serious interferences and poor signal-to-noise ratios were observed with time-domain 2*f* signals demodulated by lock-in amplifiers when modulation frequencies of 16,356.18 and 16,356.78 Hz were utilized for CH_4_ and CO_2_ detection with an interval of 0.6 Hz. To retrieve accurate gas concentrations, frequency-domain 2*f* signals were investigated using a real-time frequency analyzer with a resolution of 0.125 Hz. With the employment of multiple linear regressions, small deviations within 2.5% and good linear relationships for gas detection were observed under the frequency-division multiplexing mode with a 0.6 Hz interval of modulation frequencies. Detection limits of 0.6 ppm for CH_4_ and 2.9 ppm for CO_2_ were obtained with the analysis of the signal-to-noise ratio of the frequency-domain 2*f* signal. For the frequency-domain detection method, an additional frequency analyzer is essential for signal processing. However, a frequency analyzer can retrieve all 2*f* signals simultaneously under the frequency-division multiplexing mode when multiple gases are detected. With the 0.6 Hz interval, the amplitudes of QEPAS signals will increase substantially since the modulation frequencies are closer to the resonant frequency of the QTF. Additionally, more kinds of target gases can be measured simultaneously under the frequency-division multiplexing mode with the limited bandwidth of the QTF due to the narrow interval. Furthermore, the narrow interval of modulation frequencies can also realize frequency-division multiplexing detection for QEPAS sensors under low pressure despite the ultra-narrow bandwidth of the QTF. Future work will focus on continuous monitoring applications with this method to examine its reliability and accuracy further.

## Data Availability

The data that support the plots within this paper are available from the corresponding author on request basis.

## References

[1] Milde T., Hoppe M., Tatenguem H., Rohling H., Schmidtmann S., Honsberg M., Schade W., Sacher J. (2021). QEPAS sensor in a butterfly package and its application. Appl. Opt..

[2] Zhang H., Jin W.L., Hu M.P., Hu M., Liang J.Q., Wang Q. (2021). Investigation and Optimization of a Line-Locked Quartz Enhanced Spectrophone for Rapid Carbon Dioxide Measurement. Sensors.

[3] Levy R., Duqesnoy M., Melkonian J.M., Raybaut M., Aoust G. (2020). New Signal Processing for Fast and Precise QEPAS Measurements. IEEE Trans. Ultrason. Ferroelectr. Freq. Control.

[4] Sampaolo A., Yu C., Wei T.T., Zifarelli A., Giglio M., Patimisco P., Zhu H., Zhu H.M., He L., Wu H.P. (2021). H_2_S quartz-enhanced photoacoustic spectroscopy sensor employing a liquid-nitrogen-cooled THz quantum cascade laser operating in pulsed mode. Photoacoustics.

[5] Ma Y.F. (2020). Recent advances in QEPAS and QEPTS based trace gas sensing: A review. Front. Phys..

[6] Yi H.M., Chen W.D., Sun S.W., Liu K., Tan T., Gao X.M. (2012). T-shape microresonator-based high sensitivity quartz-enhanced photoacoustic spectroscopy sensor. Opt. Express.

[7] Dong L., Wu H.P., Zheng H.D., Liu Y.Y., Liu X.L., Jiang W.Z., Zhang L., Ma W.G., Ren W., Yin W.B. (2014). Double acoustic microresonator quartz-enhanced photoacoustic spectroscopy. Opt. Lett..

[8] Johannes P.W., Harald M., Bernhard L. (2016). Compact quantum cascade laser based quartz-enhanced photoacoustic spectroscopy sensor system for detection of carbon disulfide. Opt. Express.

[9] Giglio M., Zifarelli A., Sampaolo A., Menduni G., Elefante A., Blanchard R., Pfluegl C., Witinski M.F., Vakhshoori D., Wu H.P. (2020). Broadband detection of methane and nitrous oxide using a distributed-feedback quantum cascade laser array and quartz-enhanced photoacoustic sensing. Photoacoustics.

[10] Wang Z., Wang Q., Ching J.Y., Wu J.C., Zhang G.F., Ren W. (2017). A portable low-power QEPAS-based CO_2_ isotope sensor using a fiber-coupled interband cascade laser. Sens. Actuators B Chem..

[11] Li Y., Wang R.Z., Tittel F.K., Ma Y.F. (2020). Sensitive methane detection based on quartz-enhanced photoacoustic spectroscopy with a high-power diode laser and wavelet filtering. Opt. Lasers Eng..

[12] Kosterev A.A., Mosely T.S., Tittel F.K. (2006). Impact of humidity on quartz-enhanced photoacoustic spectroscopy based detection of HCN. Appl. Phys. B.

[13] Ma Y.F., Yu X., Yu G., Li X.D., Zhang J.B., Chen D.Y., Sun R., Tittel F.K. (2015). Multi-quartz-enhanced photoacoustic spectroscopy. Appl. Phys. Lett..

[14] Dong L., Wright J., Peters B., Ferguson B.A., Tittel F.K., McWhorter S. (2012). Compact QEPAS sensor for trace methane and ammonia detection in impure hydrogen. Appl. Phys. B.

[15] Kosterev A., Dong L., Thomazy D., Tittle F.K., Overby S. (2010). QEPAS for chemical analysis of multi-component gas mixtures. Appl. Phys. B.

[16] Zhang Q.D., Chang J., Cong Z.H., Sun J.C., Wang Z.L. (2018). QEPAS sensor for simultaneous measurements of H_2_O, CH_4_, and C_2_H_2_ using different QTFs. IEEE Photonics J..

[17] Wu H.P., Yin X.K., Dong L., Pei K.L., Sampaolo A., Patimisco P., Zheng H.D., Ma W.G., Zhang L., Yin W.B. (2017). Simultaneous dual-gas QEPAS detection based on a fundamental and overtone combined vibration of quartz tuning fork. Appl. Phys. Lett..

[18] Liu H., Hu M., Chen X., Deng H., Xu Z.Y., Wang Q., Li X., Kan R.F., Zhang X.Y. (2021). Sensitive Detection of CH_4_ and CO_2_ Using Frequency-Division-Multiplexing Based Quartz-Enhanced Photoacoustic Spectroscopy. Acta Opt. Sin..

[19] Liu Y.H., Lin H.Y., Montano B., Zhu W.G., Zhong Y.C., Kan R.F., Yuan B., Yu J.H., Shao M., Zheng H.D. (2022). Integrated near-infrared QEPAS sensor based on a 28 kHz quartz tuning fork for online monitoring of CO_2_ in the greenhouse. Photoacoustics.

[20] Wang Q., Wang Z., Ren W., Patimisco P., Sampaolo A., Spagnolo V. (2018). Fiber-ring laser intracavity QEPAS gas sensor using a 7.2 kHz quartz tuning fork. Sens. Actuators B Chem..

[21] Yi H.M. (2012). Theoretical and Experimental Research of Quartz-Enhanced Photoacoustic Spectroscopy Technique. Ph.D. Thesis.

[22] Wei M., Liu J.G., Kan R.F., Wang W., Yao L., Xu Z.Y., Yuan S., Dai Y.H., Jia L.Q. (2014). Study on Detection of Greenhouse Gases Based on Quantum Cascade Laser. Acta Opt. Sin..

